# Assessment of honking impact on traffic noise in urban traffic environment of Nagpur, India

**DOI:** 10.1186/s40201-015-0164-4

**Published:** 2015-02-12

**Authors:** Ritesh Vijay, Asheesh Sharma, Tapan Chakrabarti, Rajesh Gupta

**Affiliations:** Environmental System Design and Modeling Division, CSIR-NEERI, Nagpur, 440020 Maharashtra India; Civil Engineering Department, VNIT, Nagpur, 440010 Maharashtra India

**Keywords:** Honking, Traffic noise, Vehicular speed, Traffic volume

## Abstract

**Background:**

In context of increasing traffic noise in urban India, the objective of the research study is to assess noise due to heterogeneous traffic conditions and the impact of honking on it.

**Method:**

Traffic volume, noise levels, honking, road geometry and vehicular speed were measured on national highway, major and minor roads in Nagpur, India.

**Results:**

Initial study showed lack of correlation between traffic volume and equivalent noise due to some factors, later identified as honking, road geometry and vehicular speed. Further, frequency analysis of traffic noise showed that honking contributed an additional 2 to 5 dB (A) noise, which is quite significant. Vehicular speed was also found to increase traffic noise. Statistical method of analysis of variance (ANOVA) confirms that frequent honking (p < 0.01) and vehicular speed (p < 0.05) have substantial impact on traffic noise apart from traffic volume and type of road.

**Conclusions:**

The study suggests that honking must also be a component in traffic noise assessment and to identify and monitor “No Honking” zones in urban agglomerations.

## Background

Noise pollution, a by-product of urbanization and industrialization, is now recognized as a major problem in urban areas with many adverse health effects [1-4]. The most important factors raising noise pollution in urban areas are vehicular traffic, railway and air traffic [5,6]. Vehicular traffic contributes to about 55% of the total urban noise [7-9]. The need for studies regarding urban noise pollution and its consequences on the environment has motivated various researchers in several counties including India [10-12]. Most cities in India have been facing serious noise pollution problems in the last few decades due to substantial growth in the number of vehicles, expansion of road network, industrialization and urbanization [13-15].

Assessment of traffic noise pollution is not easy and varies with types and physical conditions of vehicles, speed, honking and road geometry [16,17]. Estimation of traffic noise is more difficult in Indian cities considering the heterogeneity in traffic conditions including mixed vehicle types, congestion, road conditions, frequent honking and lack of traffic sense [18-20]. Honking is a common occurrence in India, irrespective of road types and condition, traffic etc. [21]. Driving attitude which includes impatience, over accelerating, sudden braking, abiding traffic rules etc. may also aggravate honking. Kalaiselvi and Ramachandraiah found that horn noise events increase equivalent noise level (L_eq_) 2 to 13 dB(A) [18,21]. Therefore, there is a need to consider such diverse factors in monitoring and assessment of traffic noise as well as planning of noise abatement measures. The objective of the study is to assess and quantify traffic noise and the impact of honking on it in the urban environment of Nagpur, India. The study will help in defining new ‘No Honking’ zones in addition to assessing traffic noise and existing horn prohibited areas.

## Material and method

The methodology of the present study is elaborated in following sections.

### Study area

**Figure 1:**
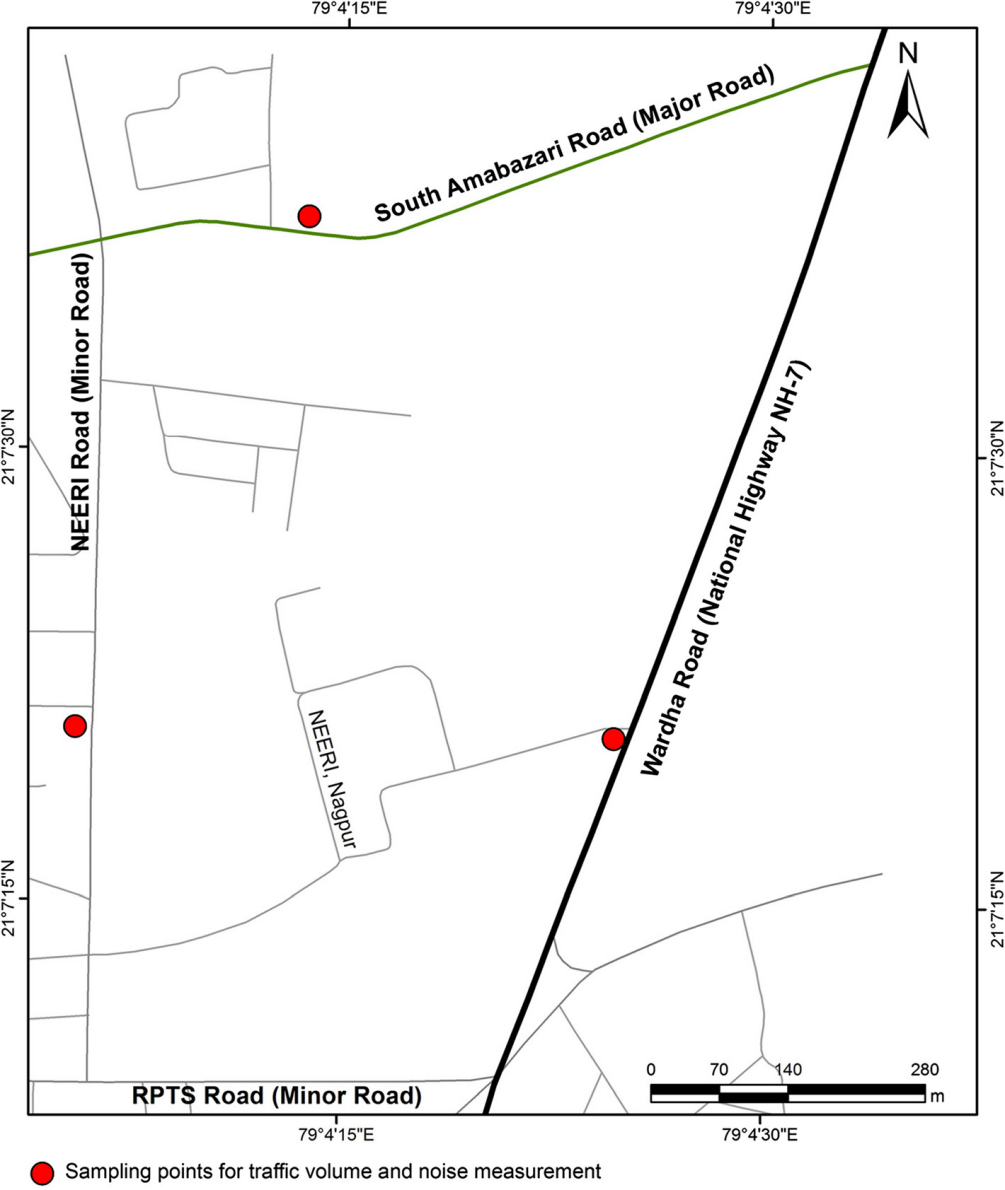
**Study area and locations for noise and traffic volume survey.**

Traffic volume, noise levels, spot speed and honking were measured at three sampling locations in the study area during March 2010 – December 2010. The study area lies between 21° 7’ 0” to 21° 7’ 45” N latitude and 79° 4’ 0” to 79° 4’ 45” E longitude in Nagpur City, Maharashtra, India (Figure 1). The study area comprises of three main roads namely Wardha road, South-Ambazari road and NEERI road. These are classified as national highway, major and minor roads respectively. Road details including geometry, category, number of traffic lanes and road conditions are considered in the study. The width of national highway, major and minor roads is 21 m, 15 m and 7 m respectively. Road conditions were almost same for all roads with asphalt surface and footpaths on both sides. Road divider separates the flow of mixed traffic at highway (six lanes) and major road (four lanes) whereas minor road doesn’t have any divider.

### Data collection

Traffic volume studies were conducted to determine the number, movements, and classification of vehicles at a given location and sampling period. Traffic volume was recorded using video camera and vehicles were counted by viewing recorded footages from cameras on computer system. Vehicles were classified as heavy (truck, bus, bulldozer, trailer, dumper), medium (car, jeep, auto-rickshaw, loading rickshaw) and light (motorcycle, scooter) based on their size and noise emission level. Auto-rickshaw is a three wheeler used as a common means of transportation in India. Noise emitted by traffic vehicles was measured as per standard methods [22,23] using sound level meter [24]. Sound level meter was mounted on a tripod stand 1.5 m above ground level with slow response mode, frequency weighting “A” and data logging of 1 second time interval. Traffic noise was measured using sound level meter at a distance of 12 m, 10 m and 5 m from the center of national highway, major and minor roads respectively. Similarly, speedometer (Speedet Traffic Radar) was mounted on tripod stand for monitoring speed of vehicles [25]. Noise emitted from a particular vehicle with corresponding speed was also measured and analyzed for noise-speed response.

### Data analysis

An attempt has been made to analyze traffic volume, vehicle speed and honking with their corresponding noise levels. Initially, traffic volume was monitored for 24 hours to identify peak traffic hours in morning and evening. Later, two sets of traffic volume and noise data were monitored during morning and evening peak traffic hours. In the first set of data, traffic and noise levels were measured for 1 hour with 15 minutes time interval while in the second set, honking along with traffic and noise level were measured for 15 minutes with time interval of 1 minute duration. Measured noise data in two sets of readings were analyzed for equivalent (L_eq_), minimum (L_min_) and maximum (L_max_) noise levels. L_eq_ was further analyzed in each time step to assess the impact of honking using frequency component of traffic noise recorded in sound level meter [26]. A statistical analysis was performed to assess the impact of diverse conditions on traffic noise based on the relationship between traffic volume, road geometry and noise data [27]. For this, analysis of variance (ANOVA) and correlation analysis were carried out to quantify the dependence of traffic volume - equivalent noise, honking - equivalent noise and vehicular speed - corresponding noise level.

## Results

Based on the analysis of 24-hour traffic volume, peak traffic flows were observed between 10:00 and 11:00 in case of highway and between 9:00 and 10:00 for major and minor roads in the morning. The number of light, medium and heavy vehicles passing through the highway were 3605, 1427 and 171, respectively during morning peak hour. The observed light, medium, and heavy vehicles on major road were 2338, 612 and 11, respectively while on minor road these values were 1587, 585 and 9, respectively. Similarly, peak traffic flow was observed between 18:00 and 19:00 for all categories of roads in the evening. Number of light, medium and heavy vehicles were 3552, 1663 and 138 at highway, 1861, 754 and 27 at major road and 1528, 611 and 8 at minor road, respectively.

**Figure 2:**
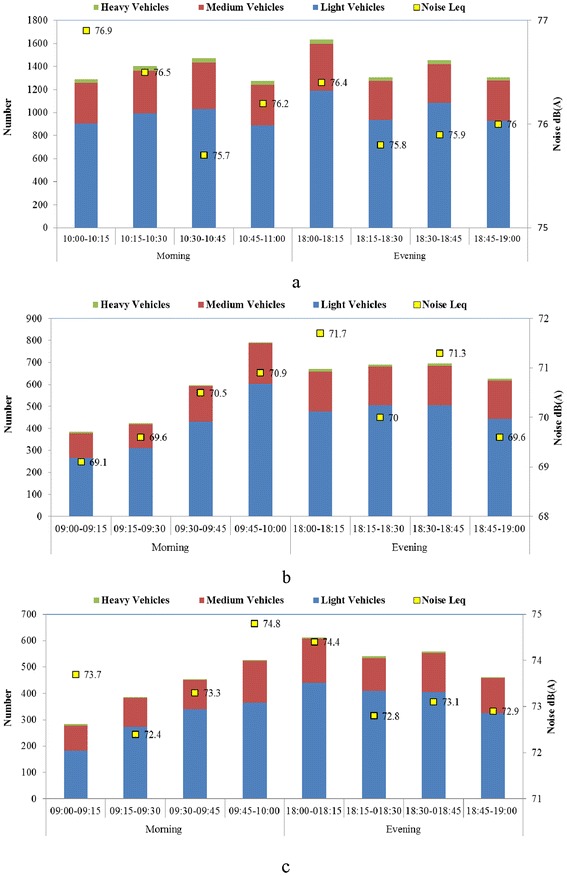
**First set of data for traffic and noise during morning and evening peak hours a) National highway b) Major road and c) Minor road.**

To assess the impact of traffic on noise levels, peak hour’s traffic and noise levels were measured for 15 minutes interval (Figure 2a, b and c) in first set of data. As per reviewed literature, noise is directly proportional to traffic volume which means that traffic noise increases with increase in traffic volume [28]. However some conflicting results were observed in the present study. For example, at highway, lowest L_eq_ [75.7 dB(A) during 10.30 to 10.45] was noted corresponding to maximum traffic volume and highest L_eq_ [76.9 dB(A) during 10.00 to 10.15] was not corresponding to maximum traffic volume during morning hour (Figure 2a); at major road, highest L_eq_ did not correspond to maximum traffic volume in evening peak hour (Figure 2b) and at minor road, lowest L_eq_ did not correspond to minimum traffic volume in morning and evening (Figure 2c). However, L_eq_ observations conformed to literature findings at highway for highest and lowest L_eq_ during evening peak hour (Figure 2a), at major road for highest L_eq_ during morning and lowest L_eq_ during evening (Figure 2b) and at minor road for highest L_eq_ in morning and evening (Figure 2c). These results show mixed trends between traffic volume and equivalent noise.

**Figure 3:**
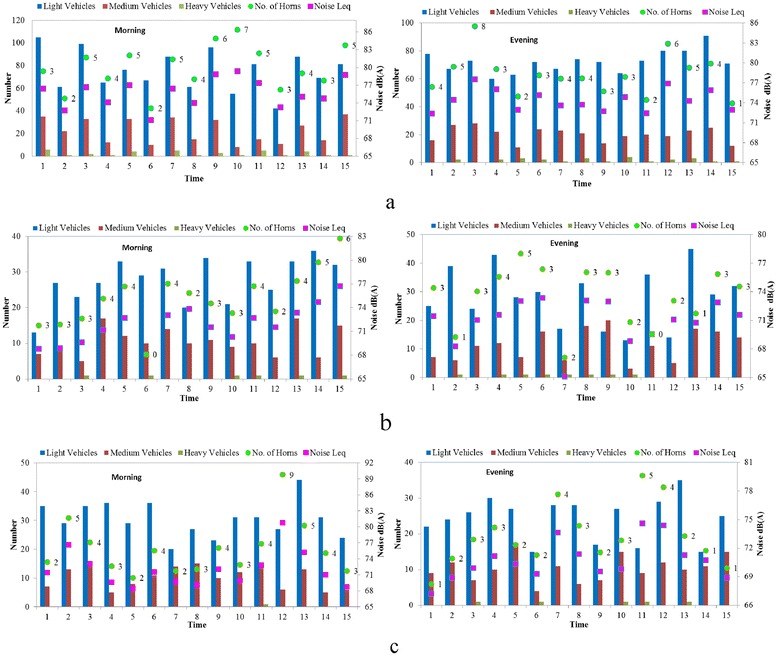
**Second set of data for traffic, noise and horn honking for 15 minutes during morning and evening peak hours a) National highway b) Major road and c) Minor road.**

As per aforementioned discussion, no statistical relationship could be found between traffic volume and noise level. This suggests that besides traffic volume, other factors are also responsible for contributing noise [19]. To identify factors responsible for traffic noise assessment, a second set of data comprising of equivalent noise, traffic volume and honking was collected. These data were collected for 15 minutes duration with one minute time interval in peak traffic hours (Figure 3). Highest L_eq_ [79.4 dB(A)] was observed in 10^th^ minute for least number of vehicles (Figure 3a) during morning at highway. This was due to maximum number of honking recorded. The maximum traffic volume was recorded in 1^st^ minute even though its L_eq_ [76.4 dB(A)] was not the highest. Although traffic volume recorded in 15^th^ minute was lesser than 1^st^ minute, noise level was more due to more number of honking. Further, for same number of horns, noise level in 2^nd^ minute was more than 6^th^ minute due to heavy vehicle. Similar results were observed in the case of 1^st^ and 14^th^ minutes. For same traffic volume in 6^th^ and 8^th^ minutes, L_eq_ was higher in 8^th^ minute due to combined effect of heavy vehicle and honking. Similar scenario of traffic noise was observed in evening peak hour at highway. Highest L_eq_ [77.5 dB(A)] was observed due to eight honking incidents recorded in 3^rd^ minute although traffic volume was not maximum (Figure 3a).

In case of major road, highest L_eq_ [76.7 dB(A)] was observed for 15^th^ minute with most honking while lowest L_eq_ [68.07 dB(A)] was observed in 6^th^ minute with no honking in the morning (Figure 3b). For same number of honks and traffic volume in 4^th^ and 11^th^ minutes, L_eq_ in 11^th^ minute was more due to presence of heavy vehicle. Though L_eq_ levels during 5^th^ and 7^th^ minutes were different, same number of horn incidents and traffic volume was observed. This variation may have been due to vehicle type, its physical condition and speed. Some contrasting results were observed at 5^th^ and 6^th^ minutes during evening (Figure 3b). For example, highest L_eq_ was observed in 6^th^ minute even though horn incidents were not recorded maximum.

Traffic and noise data on minor road during morning indicate that highest L_eq_ [80.8 dB(A)] was observed in 12^th^ minute with maximum number of horn incidents although traffic volume was not maximum (Figure 3c) while lowest L_eq_ [68.4 dB(A)] was observed in 5^th^ minute with least number of horn incidents. Further, noise level was more in 11^th^ minute as compared to 6^th^ minute with same number of honking and traffic volume due to the presence of heavy vehicle. In evening peak hour, highest L_eq_ [74.6 dB(A)] was observed at 11^th^ minute with maximum number of horns (Figure 3c).

## Discussion

**Figure 4:**
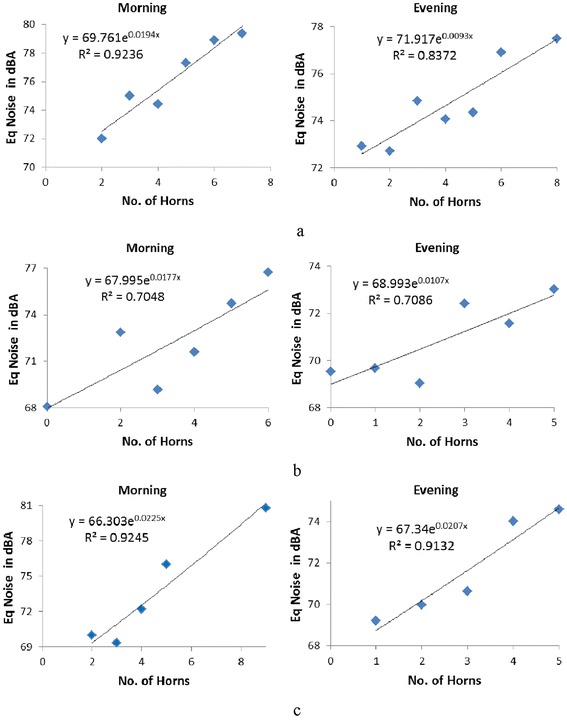
**Relationship between horn honking and equivalent traffic noise during morning and evening peak hours a) National highway b) Major road and c) Minor road.**

Second set of data suggests that honking and heavy vehicles moving on the roads have significant impact on traffic noise as compared to light and medium vehicles. In order to assess the impact of honking on traffic noise, L_eq_ and number of horns were plotted for each category of roads (Figure 4a to c). Average equivalent noise was calculated where equal number of horns was observed for every time step. A strong correlation was observed at highway and minor road while moderate relationship was observed at major road. The correlation coefficients were in the range of 0.84 to 0.97 (p < 0.05) suggesting that honking has significant impact on traffic noise, besides traffic volume. Some contradictory results were observed for some time steps where lesser number of horns produced more noise. This requires further analysis.

Further, for quantification of sound level due to honking, Type-I sound level meter was used to measure traffic noise under different frequency components distributed in the highest and lowest octaves at 16 Hz and 16 kHz in eleven octaves. Response of honking was observed mostly in the octaves of 500 Hz, 2 kHz and 4 kHz. The logarithmic addition of eleven octaves except these three octaves provides the traffic noise without honking [25]. Honking is responsible for an additional noise of 4 to 5 dB(A) during morning and 2 to 4 dB(A) in evening hour over and above traffic noise for each category of roads which was confirmed using two-way ANOVA as per Table 1. p < 0.01 for both independent variables i.e. honking and types of road, indicates that there is a significant impact of these variables on the response of traffic noise level [Confidence Interval (CI) 95%]. A comparison for traffic noise with and without honking was carried out based on the statistical data analysis (Figure 4) and data estimated using frequency analysis as presented in Table 2. Both the analyses confirm the impact of honking on traffic noise.

**Table 1:** **Analysis of variance for honking and type of road on traffic noise**

**Parameters**	**Degree of freedom**	**Sum of squares**	**Mean square**	**F**	**p**
Honking	1	42.2	42.2	72.8	0.001
Type of road	2	36.8	18.4	31.8	0.001
Interaction	2	0.4	0.2	0.3	0.735
Error	6	3.5	0.6		
Total	11	82.8			

S = 0.7; R^2^ = 95.8%.

**Table 2:** **Equivalent noise without honking as per statistical and frequency analysis**

**Type of road**	**Traffic noise L**_**eq**_ **dB(A)**		**Honking (no)**		**L**_**eq**_ **dB(A) without honking**			
					**Statistical**		**Frequency**	
	**Morn**	**Even**	**Morn**	**Even**	**Morn**	**Even**	**Morn**	**Even**
National Highway	76.6	74.7	63	57	69.8	71.9	72.2	72.1
Major	72.4	71.4	50	37	68.0	69.0	68.1	68.2
Minor	73.6	71.2	57	38	66.3	67.3	69.4	68.9

Morn – morning, even - evening.

**Figure 5:**
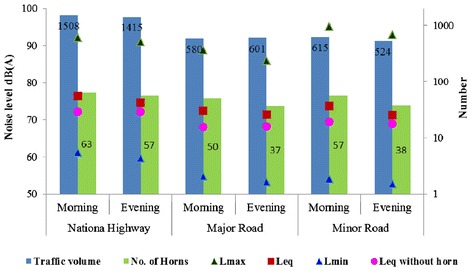
**Summarized 15-minutes traffic volume, horn and noise levels.**

The results of 15 minutes traffic and noise measurements including traffic volume, number of horns, noise levels L_min_ L_max_ and L_eq_ for with and without honking cases are summarized in Figure 5. The noise values are plotted on primary y-axis and traffic volume with number of horns is plotted on secondary y-axis while timings of measurement are represented on x-axis for each category of roads. Highest L_eq_ [76.3 dB(A)] was observed at highway with maximum traffic volume (1508) and most number of horn incidents (63 nos.). The L_eq_ at minor road is observed more than major road though traffic volume and number of horns are nearly same during peak hours. This variation is mostly due to lesser width of minor road (7 m) as compared to major road (15 m) resulting in reduction of distance between center line of road and position of sound level meter. Moreover, minor road doesn’t have divider to control the mixed traffic flow.

**Figure 6:**
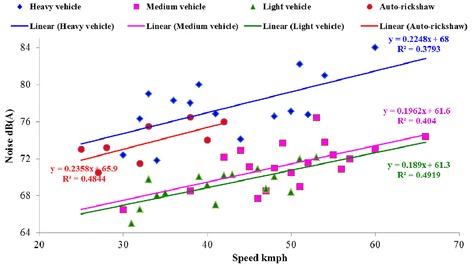
**Relationship between vehicular speed and noise level.**

A separate study was carried out to estimate the impact of vehicle type and speed on traffic noise level (Figure 6). The speed of light, medium and heavy vehicles varied in the range of 40–45 kmph, 50–56 kmph and 30–38 kmph, respectively. For all categories of vehicles, noise level varies linearly with speed. Impact of heavy vehicles and auto-rickshaw on traffic noise is comparatively more than light and medium vehicles. An increase in speed from 35 to 55 kmph, increases the noise level by nearly 4–5 dB(A) except for auto-rickshaw. While in case of auto-rickshaw, increase in speed from 25 to 40 kmph increases noise by nearly 4 dB(A). A statistical analysis using two-way ANOVA was performed to assess the significance of individual vehicle type and speed on traffic noise (Table 3). p < 0.01 for vehicular type indicates that there is significant difference in the type of vehicle on the response of traffic noise level (CI - 95%). Similarly, p < 0.05 for vehicular speed signifies the impact of variation in speed on traffic noise level (CI 95%). The analysis suggests that type of vehicle (i.e. heavy, medium, light and auto) is more dominant than vehicular speed.

**Table 3:** **Analysis of variance for vehicular type and speed on traffic noise**

**Parameters**	**Degree of freedom**	**Sum of squares**	**Mean square**	**F**	**p**
Vehicle type	3	156.9	52.3	22.38	0.001
Vehicular speed	3	31.1	10.4	4.44	0.035
Error	9	21.0	2.3		
Total	15	209.1			

S = 1.5; R^2^ = 89.94%.

## Conclusions

Monitoring and assessment of traffic noise in urban environment is complex due to various influencing factors such as traffic volume, honking, vehicular speed, road geometry etc. Traffic noise was assessed in the urban agglomeration of Nagpur, India considering above factors. Impact of heavy vehicles on traffic noise was more as compared to light and medium vehicles. Honking is a frequent phenomenon in Indian road context therefore it was observed that honking has significant impact on traffic noise besides traffic volume and vehicular speed. Previous studies also confirmed the effect of honking on traffic noise [18,21,26,29,30] and used as one of the input parameter in traffic noise prediction [31,32]. These studies do not provide quantification of honking noise in heterogeneous traffic while present research provides quantification of noise due to honking based on frequency analysis of traffic noise. This was also confirmed by statistical analysis considering traffic noise and honking data. Using this, it was found that honking induced an additional 2 to 5 dB(A) noise over and above traffic noise. Further, increase in vehicular speed from 35 to 55 kmph also increases traffic noise by 4 to 5 dB(A) for all types of vehicles. The present study suggests that honking must also be a component, apart from monitoring of traffic volume and vehicular speed in traffic noise assessment. Additionally, the study will help in assessing existing horn prohibited areas and defining new ‘No Honking zones.
